# A wide field-of-view, modular, high-density diffuse optical tomography system for minimally constrained three-dimensional functional neuroimaging

**DOI:** 10.1364/BOE.394914

**Published:** 2020-07-09

**Authors:** Hubin Zhao, Sabrina Brigadoi, Danial Chitnis, Enrico De Vita, Marco Castellaro, Samuel Powell, Nicholas L. Everdell, Robert J. Cooper

**Affiliations:** 1 DOT-HUB, Biomedical Optics Research Laboratory, Department of Medical Physics and Biomedical Engineering, University College London, London, WC1E 6BT, UK; 2 Department of Developmental and Social Psychology, University of Padova, Padova, Italy; 3 Department of Information Engineering, University of Padova, Padova, Italy; 4 School of Engineering, Institute for Integrated Micro and Nano Systems, University of Edinburgh, Edinburgh, EH9 3FF, UK; 5 Department of Biomedical Engineering, School of Biomedical Engineering & Imaging Sciences, King’s College London, London, SE1 7EH, UK; 6 Padova Neuroscience Center, University of Padova, Padova, Italy; 7 Department of Electrical and Electronic Engineering, Faculty of Engineering, University of Nottingham, Nottingham, NG7 2RD, UK

## Abstract

The ability to produce high-quality images of human brain function in any environment and during unconstrained movement of the subject has long been a goal of neuroimaging research. Diffuse optical tomography, which uses the intensity of back-scattered near-infrared light from multiple source-detector pairs to image changes in haemoglobin concentrations in the brain, is uniquely placed to achieve this goal. Here, we describe a new generation of modular, fibre-less, high-density diffuse optical tomography technology that provides exceptional sensitivity, a large dynamic range, a field-of-view sufficient to cover approximately one-third of the adult scalp, and also incorporates dedicated motion sensing into each module. Using in-vivo measures, we demonstrate a noise-equivalent power of 318 fW, and an effective dynamic range of 142 dB. We describe the application of this system to a novel somatomotor neuroimaging paradigm that involves subjects walking and texting on a smartphone. Our results demonstrate that wearable high-density diffuse optical tomography permits three-dimensional imaging of the human brain function during overt movement of the subject; images of somatomotor cortical activation can be obtained while subjects move in a relatively unconstrained manner, and these images are in good agreement with those obtained while the subjects remain stationary. The scalable nature of the technology we described here paves the way for the routine acquisition of high-quality, three-dimensional, whole-cortex diffuse optical tomography images of cerebral haemodynamics, both inside and outside of the laboratory environment, which has profound implications for neuroscience.

## 1. Introduction

Over the last 25 years, the application of functional neuroimaging technologies, particularly functional magnetic resonance imaging (fMRI), has yielded extensive and detailed insights into the relationships between brain structure, brain function and our interactions with the external world [1–3]. However, the limitations of functional neuroimaging technologies have meant that almost all of these insights are predicated on the assumption that brain function does not fundamentally differ between the laboratory and everyday environments. The ability to reliably image human brain function outside of the laboratory, using naturalistic stimuli and particularly while subjects move freely, would allow neuroscientists to investigate the effects of experimental environment and examine whole new aspects of human cognition.

Optical neuroimaging approaches such as functional near-infrared spectroscopy lend themselves to applications that are either extremely challenging or impossible with traditional neuroimaging technologies like fMRI [4–6]. Functional near-infrared spectroscopy (fNIRS) uses the differential absorption of near-infrared light transmitted through the tissues of the head to monitor cerebral haemodynamics [7–9]. In practice, fNIRS measurements are usually achieved by coupling multiple optical fibres to the scalp. These fibres transmit light to and from the subject to allow measurements of the changes in optical intensity between source and detector pairs (channels). Most fNIRS systems provide somewhere in the range of 1 to 50 measurement channels [7], which yield data that can be analysed channel-by-channel or converted into rudimentary two-dimensional maps with a resolution of around 30 mm [10,11].

In comparison to fMRI, fNIRS is relatively robust to subject movement [12,13], fNIRS devices are comparably small, are silent, portable, can be applied without stringent requirements on the local environment (e.g. no electromagnetic shielding is required) and are inexpensive to acquire and operate [7]. As a result, the fNIRS field has grown exponentially over the last decade [5,14].

Over the same period, there has been significant research effort focussed on extending the principles of fNIRS to provide three-dimensional, high-resolution functional optical neuroimaging [15–18]. Diffuse optical tomography (DOT) uses high numbers of near-infrared light sources and detectors to provide overlapping spatial sampling of the target object, which permits the acquisition of depth-resolved, three-dimensional images of human cerebral haemodynamics [19]. The advantages of increasing the density of the source and detector array are well-established and numerous: greater channel density provides greater lateral image resolution [20]; signal-to-noise is improved by virtue of the fact that multiple measurements are sensitive to every point within the field of view of the instrument [21]; and the wide range of source-detector distances permitted by high-density arrays provides depth sensitivity and specificity [22], minimizing the confounding effects of haemodynamic signals arising from the scalp [23–25]. However, increasing the number and density of the sources and detectors of near-infrared light that an instrument provides creates a number of challenges. The most obvious is the sheer number of optical fibres that must be coupled to the subject’s scalp to perform a measurement, which has approached ∼200 in some cases [18]. The use of optical fibres in these numbers severely limits the portability and applicability of the technique, and therefore undermines many of the key advantages of optical neuroimaging technologies that have underpinned the growth of the fNIRS field.

In recent years, fNIRS technologies have begun to advance away from optical-fibre-based systems that tether the subject to the bench and towards wearable devices [7]. There are now several wearable continuous wave (CW) fNIRS technologies that are commercially available, and these are already being applied to study moving subjects outside of the laboratory environment [7]. Despite these successes, all current wearable CW fNIRS technologies are subject to many of the same limitations as traditional, fibre-based devices: their low channel count and low sampling density means they have poor spatial resolution, no depth sensitivity and a narrow field-of-view.

In 2016, our group published the first three-dimensional, functional images of the human brain to be obtained with a fibre-less, high-density diffuse optical tomography (HD-DOT) system, which we refer to as the ‘microNTS’ (μNTS) system [26]. While this system exhibited field-leading sensitivity, it had a relatively narrow field-of-view, and a limited dynamic range that resulted in issues with detector saturation. In this paper, we describe the design of a new generation of modular HD-DOT technology, which overcomes these limitations. We also describe the application of this technology to a novel motor neuroimaging paradigm that was specifically designed to test the capabilities of our system, and its capacity for imaging human brain function during overt movement of the subject.

## 2. Methods

### 2.1 Module design

#### 2.1.1 Board layout and control unit

**Fig. 1.**
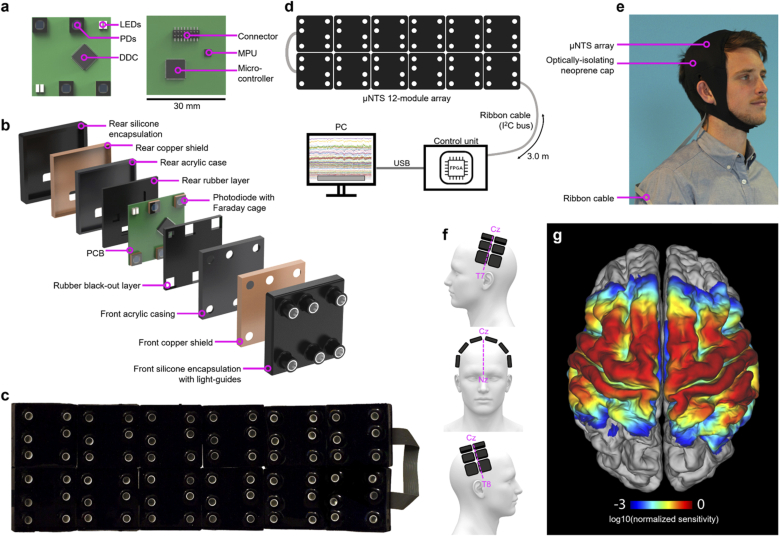
**The completed 12-module HD-DOT imaging system.** a) Photographs of both sides of the module PCB with key components indicated; b) an exploded view of the module encapsulation layers; c) a photograph of 12 modules in a 2 × 6 formation; d) a system-level diagram showing the 12 modules, control unit, PC, and cabling; e) the system in place on a subject, with neoprene cap; f) diagrams illustrating the anatomical positioning of the 12-module array on the head of our subjects; and g) the group-average sensitivity map calculated via photon transport modelling indicating the regions of the cortex to which the array was sensitive.

The full system consists of 12 independent DOT sensor modules (Fig. 1). Each module consists of a 30 mm × 30 mm 4-layer printed circuit board (PCB) (Fig. 1(a)). Two pairs of LEDs (OIS-170-740-XT, OIS-170-IT855-XT, OSA-Opto, Germany) with wavelengths of 740 nm and 855 nm were selected to act as the optical sources, and were positioned at opposite corners of each board [27,28]. Four silicon photodiodes (photosensitive area: 2.77 × 2.77 mm^2^, S12158-01CT, Hamamatsu, Japan) were used as detectors. Two photodiodes are positioned at the remaining two corners of the PCB, and two are positioned at 10 mm from the centre of each pair of LEDs to form a short-separation channel [29]. Each module therefore provides within-board source-detector separations of 10 mm (2 channels), 23 mm (4 channels) and 28 mm (2 channels). In total the system incorporates 24 source and 48 detector locations and yields 12 × 2 × 12 × 4 = 1152 NIRS channels per wavelength. Twelve modules provide coverage of approximately 220 cm^2^, which equates to around one third of the accessible scalp surface of a typical adult [27,30]. When positioned on the scalp over the midline (Fig. 1(e),(f)), a 2 × 6 formation of modules allowed us to acquire data from across the somatosensory and motor cortices of both hemispheres in our adult subjects. Figure 1(g) shows the regions of the cortex to which the 12-module system was sensitive, as determined via photon transport modelling in subject-specific anatomical finite element meshes.

Each photodiode was connected to one of the four-channels of a charge-to-digital converter (DDC114, Texas Instrument, USA), consisting of a charge integration amplifier and a 20-bit Sigma-Delta analogue-to-digital converter. The integration of the charge amplifier and the analogue-to-digital converter into the same silicon substrate within a monolithic package allows the DDC114 to provide higher signal-to-noise performance than conventional discrete components. Each channel of the DDC114 has two independent integrators with a common input. When the integrated charge is converted to a digitized measurement from one integrator, the other integrator simultaneously accumulates new charge from the photodiode. This strategy avoids dead-time and permits continuous data acquisition.

A 32-bit ARM Cortex-M0 based micro-controller unit provides local control for each module. It regulates LED source illumination, retrieves optical measurements from the DDC114 and motion sensing data from the motion processing unit (MPU) and writes that data to an Inter-Integrated Circuit (I^2^C) bus. An 8-pin connector on the rear of each board allows the connection of a ribbon cable for power and data transmission. During operation, the power consumption of each module is 160 mW. A control unit based on a Field Programmable Gate Array (FPGA) provides power and control for all modules via the single ribbon cable (Fig. 1).

#### 2.1.2 Shielding and encapsulation

While the adoption of the DDC114 allows for a high detection sensitivity, it is vulnerable to external sources of noise that include electro-magnetic interference and capacitive coupling. The principal source of electro-magnetic interference is mains power line noise. If the integration cycle does not match the power line frequency (i.e. 50 Hz in the UK), charge will be induced during each integration cycle. Due to unpredictable variations in the power line frequency, it is not possible to correct for these effects retrospectively. Capacitive coupling can also induce extra charge as a result of electrostatic build-up due to the low permittivity of the encapsulation materials. In order to eliminate the impact of these noise sources, we applied two stages of electromagnetic shielding. A dedicated copper shield was placed around each photodiode. Each shield had a 4 mm diameter circular opening on the upper surface to allow light to be coupled into the photodiode. This window was covered with a layer of Indium Tin Oxide (ITO) film to maintain the continuity of the shield while allowing for optical transmission. Each shield was connected to the ground of the system. A custom multilayer encapsulation (Fig. 1(b)) was then implemented to provide electrical isolation between the opto-electronics and the human scalp. Internal light leakage from LEDs to photodiodes was minimized by placing neoprene rubber optical isolation pads between the source and detector elements. The board was then placed inside a laser-cut acrylic case, which was itself permanently encased in another layer of copper shielding. A bespoke silicone light-guide with a diameter of 3.5 mm was used to couple light to and from the optical components through the layers of encapsulation to the scalp. Finally, a black-pigmented silicone rubber was moulded over the external shielding and around the light-guides to form a permanent, seamless, wipeable surface, with only a connector exposed on the upper surface. Including the 4 mm protrusion of each light guide, the total height in cross-section of the module is 14 mm. The weight of each module is approximately 15 g.

#### 2.1.3 Sequence and signal optimization

In order to achieve a 12-module system with a minimum frame rate of 3 Hz, all required measurements associated with a given LED had to be obtained within 6.8 ms (48 LED illuminations plus one dark measurement per frame). It was also critical to ensure that the shortest channels were not at risk of saturation during in-vivo recordings. To achieve this level of performance, we took advantage of the fact that the DDC114 is programmable in terms of both gain and integration times. The maximum charge that can be accumulated on each channel of the DDC114 was 50 pC, with a theoretical Root-Mean-Squared noise of 0.63 fC. This is equivalent to 205 fW and 1.63 nW of optical power respectively, resulting in a theoretical maximum dynamic range of 98 dB.

**Fig. 2.**
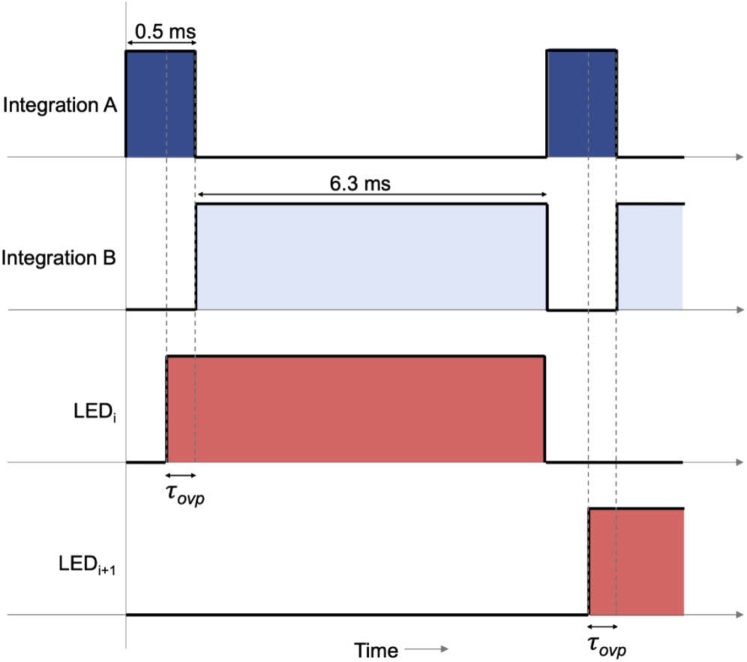
**A timing diagram of the dual integration data acquisition scheme.** Integration A corresponds to the short integration period (0.5 ms) and B, the long integration period (6.3 ms). The LED overlap time *τ_ovp_* can be optimized on a subject-by-subject basis.

However, in order to further increase the dynamic range, we implemented a dual-integration strategy, wherein both a short and a long integration period were undertaken within the 6.8 ms window available for each LED. In this dual-integration scheme, the data acquisition sequence for each LED consists of two separate integrations, labelled as A, and B in Fig. 2. The beginning of the sequence starts with the short integration (A, 0.5 ms) followed by a longer integration time (B, 6.3 ms). The respective LED is only switched on shortly before the start of the long integration, B. This is to ensure the LED is illuminated for a very short time within the integration A. The time between the rising edge of the signal driving the LED and the start of integration B is referred to as the overlap time (*τ_ovp_*), which is programmable: it can be set at any value between 0.02 ms and 0.5 ms with a 0.02 ms resolution. As part of the system initialisation procedure, in each subject users can select the maximum value of *τ_ovp_* that results in there being no saturated measurements from any short integrations. After the short integration period, as much of the time remaining in the 6.8 ms sample window as possible is allotted to the long integration. The LED remains on continuously throughout the long integration period.

The advantage of this dual-integration approach is that it has an effect equivalent to increasing the maximum detectable intensity by a factor equal to the ratio of the long integration period and the minimum value of *τ_ovp_*, which is in excess of 300. If short-separation measurements from the long integration period are found to saturate the detector, the short-integration measurement can be used instead. Typically, which integration will be used for a given channel is determined for each subject as part of initialisation of the system, with the result stored in a simple look-up table for use in post-processing.

#### 2.1.4 Motion sensor implementation

The motion sensor (MPU9250) has the potential to provide 3-axis accelerometry, 3-axis gyroscope and 3-axis magnetometry data at between 100 and 200 Hz. An I^2^C protocol was implemented to achieve two-way communication between the motion sensor and the local microcontroller within each module, which then loaded MPU data onto the data bus for transfer to the control unit. Due to limitations in data transmission rates, it was not possible to transfer all 9 axes of MPU data and simultaneously extract HD-DOT data from 12 DOT modules. A balance had to be struck between the sample rate of MPU data, the number of data axes that were transmitted per module, and the number of modules recording motion sensor data. Ultimately we chose to extract MPU data from every module at 150 Hz. To achieve this within the bandwidth limitations meant it was only possible to transmit a single axis of data per module. We chose therefore to transmit the norm of the three accelerometer axes, which we thought likely to provide the clearest indication of the occurrence of motion artifacts.

### 2.2 Array positioning and application

To couple the array to the head of the subjects, the 12 modules were first placed within a stretchable lattice made from 1 cm wide elastic strapping. The elastic ran around the outer edge of each module, and held the modules in formation while allowing the array to conform to the head. A continuation of the elastic lattice at each end of the array formed a chin-strap. Tightening the chin-strap caused the inter-module distance along the long axis of the array to increase. The minimum inter-module distance was simply the thickness of the elastic strapping (∼2 mm), while the maximum distance was limited only by the length of the ribbon cable between modules, but this limit was never approached. When applied to the subjects, the array was centred along the midline, 1 cm posterior to Cz, with the long axis of the array running parallel to the T7-Cz-T8 line (Fig. 1(b)). The chin-strap was tightened such that the centre of the lateral edges of the array reached C5 on the left and C6 on the right hemisphere. A Polhemus Patriot 3D digitizer (Polhemus, VT, USA) was used to measure each subject’s cranial landmarks (nasion, inion, pre-auricular left and right, Cz) and the external corners of each module within the array for registration purposes. Once the registration measurements were complete, a neoprene cap was applied over the module array to maintain downward pressure on the modules and insulate them from external light sources (see Fig. 1(e)). A single ribbon cable of 3 m in length connected the modules to one another and to the control unit, allowing subjects to move in a relatively unconstrained manner.

### 2.3 Experimental preparation and paradigm design

Nine subjects (2 female; median age 28 and range 22-45 years, 1 left handed) were recruited to this study. Each gave written, informed consent prior to undergoing any experiment. This study was approved by the UCL research ethics committee.

Structural magnetic resonance imaging (MRI) data were acquired for each participant using a Siemens Magnetom Prisma Fit 3T system. This included a 3D T1-weighted sequence (MPRAGE, 1 mm isotropic, TR/TE/TI = 2200/2.9/900 ms) and a 3D T2-weighted sequence (SPACE, 1 mm isotropic, TR/TE = 3200/400 ms). The MRI scans and HD-DOT experiments were performed on different days within the same 4-month period.

Prior to the start of an experimental session, the subjects were given 5 minutes to familiarize themselves with texting the pangram ‘the quick brown fox jumps over the lazy dog’ on an iPhone 7 smartphone (Apple Inc., CA, USA). The HD-DOT data acquisition experiment then consisted of three separate paradigms, which were randomly ordered for each subject and are described below.

The first paradigm was a unimanual texting protocol performed while the subjects remained (with the exception of the task itself) as still as possible. Subjects were seated in a comfortable chair, approximately 90 cm from a computer screen. Using a timing protocol built in MATLAB (Mathworks, MA, USA), the screen would initially (and during inter-stimulus ‘rest state’ periods) display a fixation cross at the centre of a 50% grey screen. To trigger the start of a stimulation block, the words ‘Left hand text’ or ‘Right hand text’ were displayed on the screen and accompanied by an audio cue of the same words read aloud. During each block, the subjects would text the pangram above continuously (without capitalization nor punctuation) for 17.5 seconds with the specified hand, at which point the block would end, as indicated by the screen reverting to the rest state and the audio cue ‘stop texting’. Inter-stimulus periods were randomly jittered in duration from 17.5 to 22.5 seconds. A total of 16 stimulation blocks (8 per hand in a pseudorandom sequence) were performed, so that the total duration of this paradigm, which including 30 seconds’ rest at the beginning and end of the sequence, was approximately 11 minutes.

The second paradigm consisted of a simple walking protocol. Subjects were asked to stand, and on hearing the audio cue of the word ‘walk’, walk at their own pace around the laboratory in a figure-eight path spanning the width of the room (approximately 3.5 m). Each walking block would last 17.5 seconds, with the end of each block indicated by the audio cue ‘stop walking’. At the end of each block, subjects would stop walking and stand still at whatever point in their path they found themselves. The inter-stimulus period was randomly jittered in duration from 17.5 to 22.5 seconds. A total of 12 stimulation walking blocks were performed, so that the total duration of this paradigm, which included 30 seconds’ rest at the beginning and end of the sequence, was approximately 8.5 minutes.

The last paradigm was a unimanual texting protocol performed while the subject walked continuously throughout. Subjects were asked to walk the figure-eight path at their own pace. To trigger the start of a stimulation block, the words ‘Left hand text’ or ‘Right hand text’ were provided by audio cue. During each block, the subjects would continue to walk, but would simultaneously text the pangram above continuously for 17.5 seconds with the specified hand. The end of each block was indicated by the audio cue ‘stop texting’. Inter-stimulus periods were randomly jittered in duration from 17.5 to 22.5 seconds. A total of 16 stimulation blocks (8 per hand) were performed, so that the total duration of this paradigm (including 30 seconds’ rest at the beginning and end of the sequence) was approximately 11 minutes.

### 2.4 HD-DOT data processing, image reconstruction and analysis

#### 2.4.1 Head modelling and array registration

For each subject, the T2-weighted image was linearly co-registered to the T1-weighted image using ANTs [31]. Both T1-w and T2-w images were employed to produce a multi-tissue probabilistic segmentation using the unified segmentation algorithm implemented in SPM12 [32] (http://www.fil.ion.ucl.ac.uk/spm/). Six tissue types were segmented: soft tissue (i.e. scalp), skull, cerebrospinal fluid (CSF), grey matter (GM), white matter (WM) and air. The final 6-layer tissue mask of each subject was obtained by assigning to each voxel the label of the tissue with the highest probability. High-resolution multi-layer tetrahedral meshes were then created with the iso2mesh software [33] for each subject.

Cranial landmarks (Inion, Nasion, pre-auricular points and Cz) were manually identified for each subject from a 3D rendering of their T1-w MRI and used to map the cranial landmark coordinates measured with the Polhemus digitizer to the virtual head mesh coordinate system via an affine transformation. The transformation matrix obtained in this process was then applied to all points measured with the Polhemus system, which included the external corners of each module. Each scalp surface mesh was then realigned to the measured positions using the iterative closest point algorithm [34]. Sources and detector positions were then obtained from a priori knowledge of their position with respect to the measured corners of the tiles, using a modified version of the spring relaxation algorithm included in the Homer2 package, AtlasViewer [35].

#### 2.4.2 Forward modelling

Wavelength-specific Jacobians were computed on the volumetric head meshes of each subject with the Toast++ software [36], which solves the diffusion approximation via the finite element method. Optical properties (absorption coefficients and reduced scattering coefficients) were assigned to each tissue type and for each wavelength by fitting all published values for these tissue types [28,37,38]. Voxels within the head that were defined by the segmentation process as air (typically within the sinuses) were assigned the same optical properties as CSF. The forward model solution was projected by Toast++ onto a 50 × 60 × 50 regular voxel grid for reconstruction, and a finer inter-mediate grid of 100 × 120 × 100 voxels was used to optimize the mapping between the mesh space and the voxel space. Sources (and adjoint detectors) were modelled as having a Gaussian profile with a standard deviation of 2 mm, and Neumann boundary conditions were applied.

#### 2.4.3 Data pre-processing

The raw, channel-wise HD-DOT data was processed prior to image reconstruction using elements taken from the Homer2 toolbox (https://homer-fnirs.org/) to produce haemodynamic response functions (HRFs) for each subject and condition. First, channels were passed through a quality control step (enPruneChannel), which removed channels that exhibited a mean intensity of less than 500 ppm or a value of the mean intensity divided by the standard deviation of less than 12. Any channels that, despite the dual-integration strategy described above, were found to saturate at any point during each recording were also excluded. This quality control step resulted in a binary vector indicating those channels that had passed these thresholds (‘good’ channels). After converting the intensity data to changes in absorbance (in units of optical density), a motion correction step was performed using the kurtosis wavelet approach with a kurtosis level of 3.3 [39]. The function hmrMotionArtifactByChannel was applied both before and after wavelet motion correction to quantify the burden of motion artifacts in both the original data and that which remained in the data after the motion correction step. This information was used to permit data rejection where necessary and to yield the artifact envelope used in the comparison with the motion sensor data of Fig. 6 in Section 3.4. Note that a modified version of the hmrMotionArtifactByChannel function was also used to identify features in the MPU data. After these motion correction steps, the data was bandpass filtered in range 0.01 to 0.5 Hz and converted to changes in haemoglobin concentrations using differential pathlength factors of 7.5 and 7 for 740 nm and 855 nm wavelengths, respectively [40]. Haemodynamic response functions were then extracted using a deconvolution approach that employed a set of gaussian basis functions (with both a standard deviation and temporal spacing of 1.5 s) to model the HRF from 5 seconds prior to 30 seconds after the onset of each stimulus [23]. For every channel in the array, the deconvolution model included as a regressor the nearest short-separation channel (defined as that with a source-detector separation of 10 mm or less) that passed the quality control step. This process yielded 35-second long haemodynamic responses for every good channel, condition and subject.

#### 2.4.4 Inverse problem

Images representing both the change in oxyhaemoglobin (ΔHbO) and the change deoxyhaemoglobin (ΔHbR) were reconstructed for each subject, task and time point using a multispectral approach, which directly generates concentration changes from attenuation data [41]. We employed the LSQR method to solve the inverse problem and first order Tikhonov regularization, as in Brigadoi et al. [42]. For each reconstruction, the LSQR algorithm was configured to terminate after a maximum of 50 iterations, and with a tolerance of 10^−5^. The regularization hyperparameter was set to λ = 10^−3^. The reconstructed images were defined on the same regular grid as the Jacobian. A remapping procedure was therefore performed to map back the reconstructed images from the voxel space to the volumetric head mesh.

#### 2.4.5 Group-level analysis

For each subject, the structural pipeline of the Human Connectome Project (HCP) [43] (HCP pipeline v. 4.0.0, https://github.com/Washington-University/HCPpipelines/releases) was implemented to obtain a pial surface in the subject-space and a high-resolution pial surface mesh with 164,000 nodes registered to the Conte-69 template [44].

For surface analysis and visualization, each volumetric head-mesh-based image was mapped to the corresponding pial surface in the subject-space by assigning a value to each node on the pial boundary surface that was equal to the mean value of all the volumetric mesh node values within a 3 mm radius. In order to compute group average images and perform statistical analyses to compare activation across tasks, the ΔHbO and ΔHbR pial images in the subject space were first resampled to the high-resolution Conte-69-registered subject-space. Group averages for both ΔHbO and ΔHbR were then computed by averaging all the Conte-69-registered pial images for each task at each reconstructed time point in the template space.

For each subject, task and chromophore, the average activation image between 6 and 16 s post stimulus-onset was computed. Since the 12 modules were designed to cover the sensorimotor cortex in this experiment, we reduced the number of nodes on which to apply statistical comparisons by selecting only nodes within the regions to which the 12 modules were sensitive. The sensitivity mask was computed by mapping to the Conte69 template the sensitivity map for the subject with the highest number of good channels, i.e. the one achieving the highest sensitivity over the cortex (our best-case scenario), and setting to 0 all nodes with sensitivity lower than 5% of the maximum sensitivity.

Statistical analyses were performed using the Permutation Analysis of Linear Models (PALM) tool included in FSL [45]. PALM is a statistical toolbox that uses permutation methods and includes support for surface-based statistical analysis. Permutation-based t-tests were performed to compare each activation image vs. rest (defined as the average image during the baseline period from -5 to 0 seconds) and to compare activation images for different tasks, with 500 permutations, tail approximation [46] and threshold-free cluster enhancement to correct for multiple comparisons with default parameters for surface data.

## 3. Results

### 3.1 System performance and data quality

By taking the standard-deviation of dark measurements over time for each detector and each experiment, it is possible to quantify the sensitivity of the system in terms of the measured noise-equivalent power (NEP) [30]. The median NEP measured in-vivo, across all 9 subjects, 3 paradigms and all 48 detectors was 318 fW (range 268 to 409 fW). A 3-way ANOVA of subject, paradigm and detector demonstrated that all three variables have a significant impact on NEP (p < 0.001), with subject having the most significant effect (p < 1 × 10^−6^). The maximum detectable power for the long integration period with the chosen gain is 16.3 nW, and as a result, the measured dynamic range was 94.2 dB. However, accounting for the increase in the effective maximum detectable power afforded by dual-integration the effective dynamic range of the system was 142.6 dB. Note that the minimum value of *τ_ovp_* automatically determined across our subject group was 0.1 ms. As this system is an evolution of previous devices, basic assessments of features such as temporal stability are beyond the remit of this paper. However, we include as supplementary data an example of a simple stability measurement (see Fig. 7 in the Appendix).

The data quality control steps described in “Methods” allowed us to quantify the proportion of ‘good’ channels obtained in each subject as a function of source-detector separation. Two subjects from the cohort were found to have less than 70% of channels with source-detector separations in range 0-35 mm marked as ‘good’, and were subsequently rejected. The median percentage of channels in range 0-35 mm marked as good in the remaining 7 subjects was 94.5%. The maximum source-detector separation of channels marked as ‘good’ varied from 34.8 to 56.5 mm across our subject group and the corresponding number of dual-wavelength channels used for image reconstruction varied from 248 to 614.

### 3.2 Functional images

**Fig. 3.**
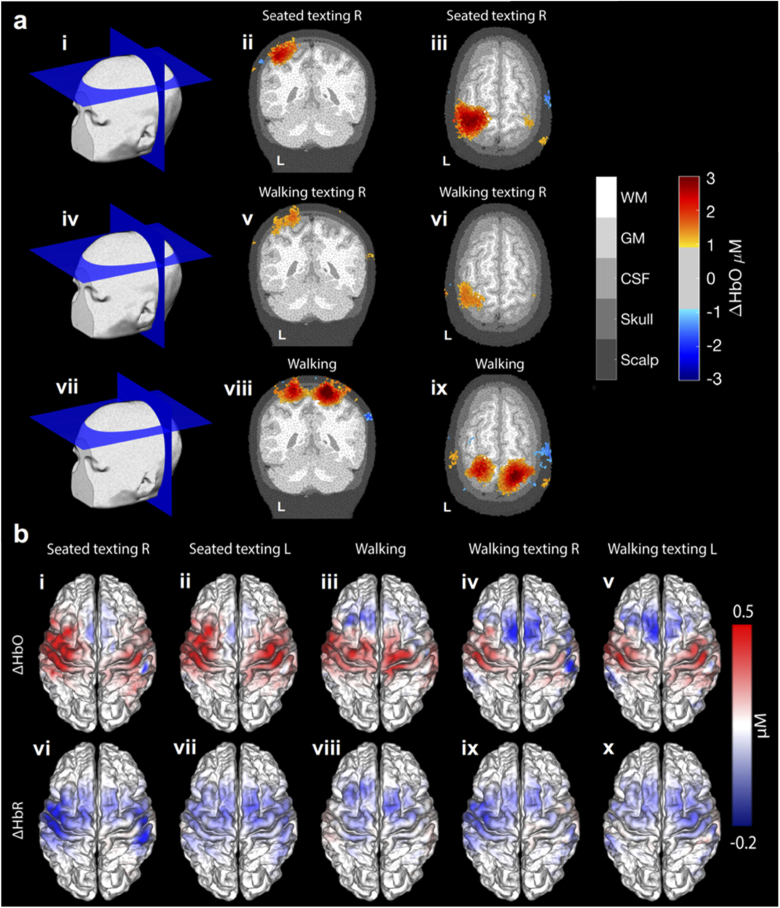
**HD-DOT haemodynamic images at the subject and group level.** Part (a) depicts example 3D orthogonal slices through peak ΔHbO response DOT maps in response to texting with the right hand while seated, texting with the right hand whilst walking, and walking, each thresholded at 30% of the peak response. Group-average haemodynamic response maps, displayed in the Conte 69 template pial space, are shown in part (b). Images b(i-v) show the ΔHbO maps for each of the five conditions, while b(vi-x) the equivalent ΔHbR images.

Example three-dimensional DOT functional response images are presented in Fig. 3(a). Reconstructed images of the peak response in oxyhaemoglobin concentration for the seated texting (right), walking texting (right) and walking paradigms are presented in a subject-specific finite element mesh. Group-level, peak-response maps associated with all five task conditions, for both ΔHbO and ΔHbR are shown in Fig. 3(b). A clear, localized contralateral response is apparent for the seated texting with the right hand, while the left-hand seated texting produces a more bilateral response pattern. In comparison the walking task produces a positive ΔHbO in the superior somatomotor cortex and only a small negative ΔHbR, predominantly in midline cortical areas anterior to the pre-central gyrus. The walking texting response maps show a positive ΔHbO and a negative ΔHbR that are similar in spatial distribution to those of the seated-equivalent, but with notably reduced contrast. Walking texting (with both left and right hand) are associated with a decrease in ΔHbO in the superior supplementary motor area relative to the baseline condition.

**Fig. 4.**
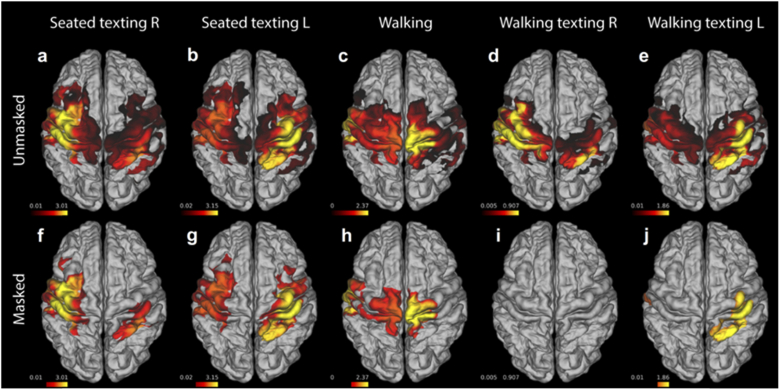
**Group T-stat maps for the contrast of task ΔHbO > baseline ΔHbO for each of the five conditions.** The upper row (a-e) consists of the unmasked T­-statistic maps, while the lower row (f-j) shows the masked T-stat maps in which only values that remain significant after correction are displayed.

Cortical maps of the T-statistic associated with the contrast of task ΔHbO > baseline ΔHbO for all five tasks are shown in Fig. 4(a-e). The same maps, but masked to indicate significance after correction at a T-stat value equivalent to p < 0.05 are shown in Fig. 4(f-j). Note that the T-stat maps of the seated and walking texting conditions are very similar in their spatial distributions, with contralateral increases in HbO apparent in each case. Significant responses are apparent in the contralateral hand-knob regions of the somatomotor cortex to both seated texting conditions. Walking texting left also demonstrates a significant response in the hemisphere contralateral to stimulation, but with a notably reduced extent. No region of the brain exhibited a significant response to the walking texting right condition, despite the similarities to the equivalent seated condition apparent in the haemodynamic maps of Fig. 3(b). The walking condition produced a significant ΔHbO response in the superior somatomotor cortices. Note that while the ΔHbR images of Fig. 3(b) show decreases that are spatially localized and generally consistent with a typical functional haemodynamic response, the T-statistic images based on ΔHbR did not reach significance after correction.

**Fig. 5.**
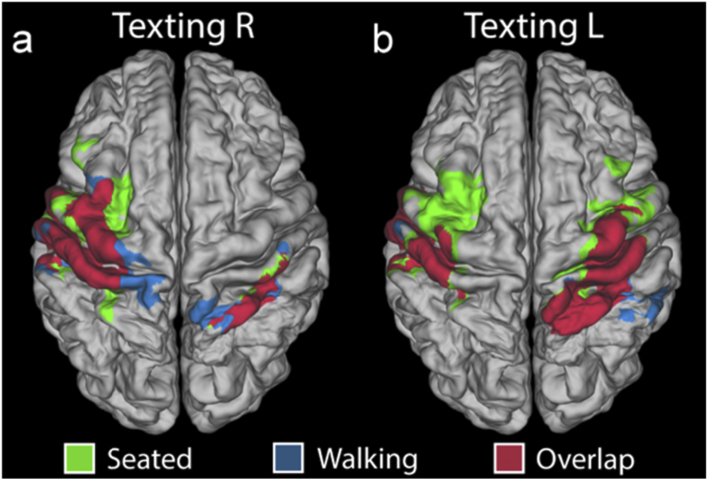
**Overlap images comparing the spatial distribution of seated and walking functional T-stat maps**. (a) shows masks of the region in which the T-stat map exceeds 30% of its maximum for seated texting right (green) and walking texting right (blue). The regions where these masks coincide is shown in dark red. The equivalent image for texting with the left hand is shown in (b).

Figure 5 depicts the spatial overlap of the ΔHbO-derived T-statistic maps for the seated and walking texting paradigms for both right and left-handed tasks, when each is thresholded at 30% of its maximum T-statistic value. Areas in which the seated texting and walking texting distributions overlap are indicated in dark red. The dice coefficient, which was calculated to quantify the extent of this overlap, was 0.77 and 0.75 for right and left-handed tasks, respectively.

### 3.3 Motion artifacts and motion sensor data

Despite offering less vulnerability to motion artifacts than other neuroimaging modalities, fNIRS is still sensitive to motion [12,13]. Motion artifacts are often a significant problem for fNIRS applications, particularly in studies of infants and in studies of moving subjects. For this study, the proportion of data identified as motion artifact was consistent across our three different experimental paradigms, despite two paradigms requiring the subjects to walk around the laboratory. The burden of motion artifact (the percentage of data-points identified as motion) averaged 4.0%, 2.2% and 2.4% for the seated texting, walking and walking texting paradigms, respectively, but these differences were not statistically significant (one-way ANOVA, p = 0.089). Similarly, the number of channels passing our data quality thresholds showed no significant variation across the three paradigms (one-way ANOVA, p = 0.996). However, the standard deviation of the absorbance data (which averaged 0.0252, 0.0262 and 0.0304 OD for the seated texting, walking and walking texted paradigms, respectively) were all significantly different from one another (one-way ANOVA, Tukey-Kramer post-hoc analysis, p < 0.01). Therefore, while the proportion of identified motion artifacts did not change in the walking paradigms, the act of walking appears to significantly increase the standard deviation of our HD-DOT data.

**Fig. 6.**
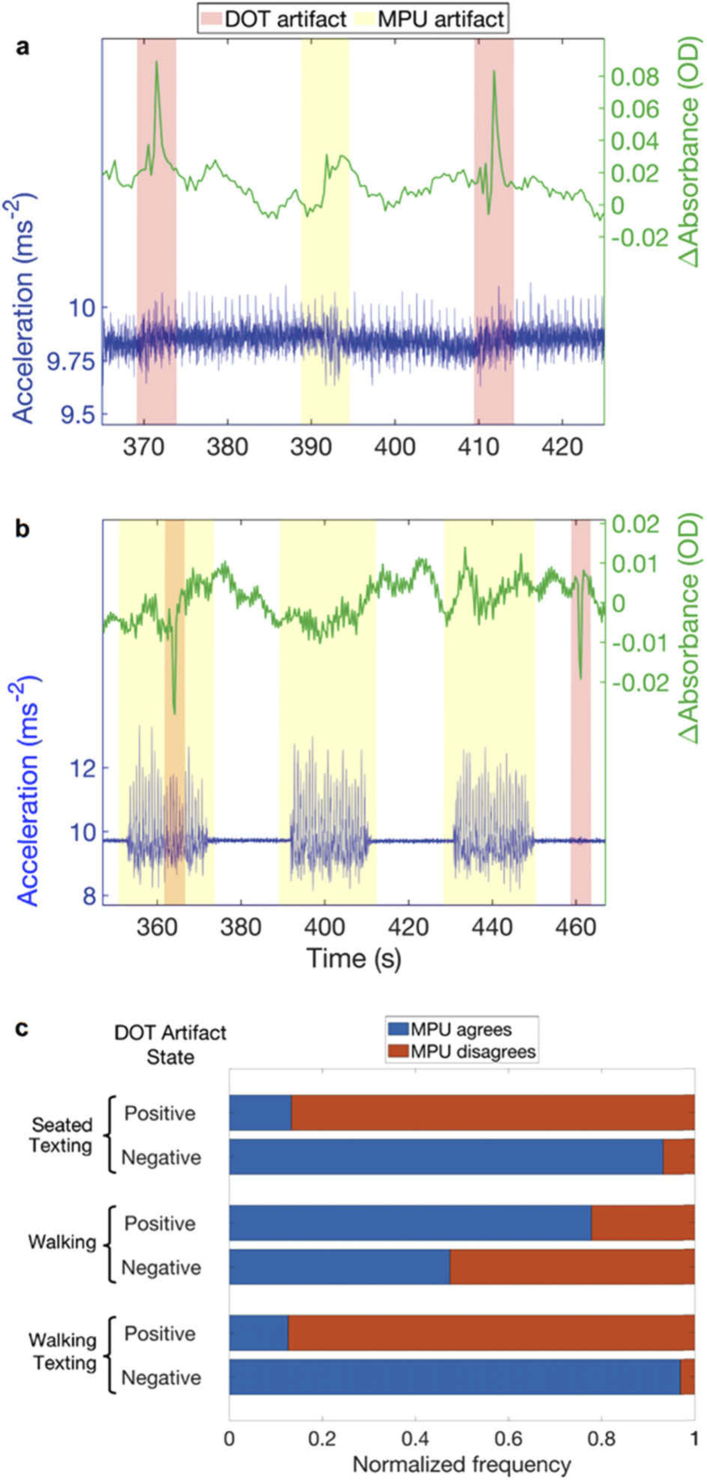
**The relationship between motion sensor data and DOT artifacts.** a) A selected period of accelerometery data from one module (in blue) and the unfiltered change-in-absorbance signal at 740 nm for a channel associated with that same module (in green). The overlaid pink shaded regions indicate periods identified as motion artifacts using the DOT data (see “Methods”) while the yellow shaded periods identify features from the MPU signal. Note the discrepancies between the two. Panel b) presents equivalent data for the walking paradigm. Note the overt periods of increased acceleration variance during the walking blocks. Motion artifacts identified in the DOT data do not coincide with detectable changes in the accelerometer signal, which appears dominated by the acceleration associated with the walking task itself. Panel c) Provides a summary of the concordance between the DOT- and MPU-derived artifact envelopes across all datasets.

**Fig. 7.**
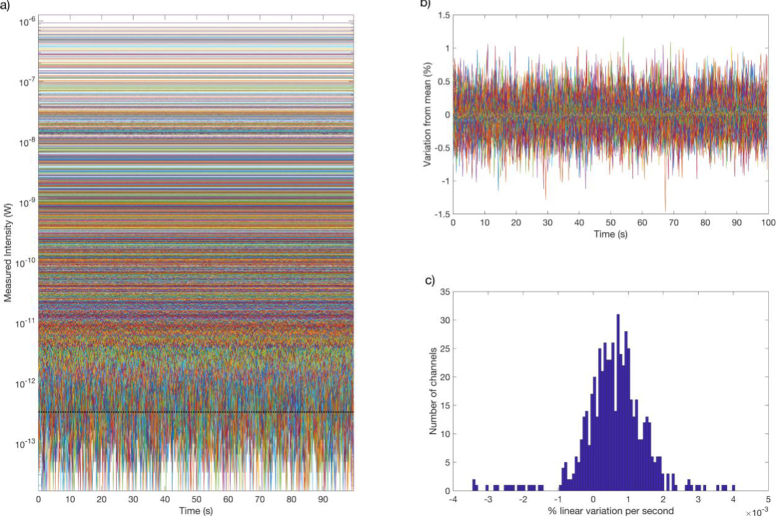
Phantom characterization of the thermal stability of the 12-module HD-DOT system.

To inform and potentially alleviate the issue of motion artifacts [47,48,56,57] (and to take further advantage of the concept of modular, wearable HD-DOT technology) a motion sensor (MPU9250, Invensense Inc. USA) was incorporated into each module. The norm of the 3-axis accelerometry data from each motion sensor was collected for all 12 modules throughout all three paradigms in each subject. Figure 6(a) shows an example period of acquired accelerometer data from one module, and an unfiltered change-in-absorbance trace from one DOT channel associated with that module, for the seated texting paradigm in one subject. The figure also displays the periods identified as potentially artifactual using the MPU signal, and the periods identified as artifact from the DOT data itself using established approaches. The clear spike-like artifacts identified directly from the DOT data do not reliably coincide with identifiable features in the accelerometer signal, even when the subject is seated. Figure 6(b) shows an example period of data from the walking paradigm. Here, the walking blocks are clearly evident in the MPU data. Here, even large spike artifacts in the DOT data do not coincide with clear deviations in the MPU data while the subject is walking. Similar patterns are evident throughout the dataset. Figure 6(c) summarises the agreement between the DOT-derived artifact envelope and the MPU-derived equivalent. For the seated and walking texting cases, when an artifact is apparent from the DOT data, the MPU rarely agrees (rates of only 13.3 and 12.6% respectively). When there is no artifact identified in the DOT data (which is the vast majority of the time) there is greater agreement between the DOT and MPU-derived envelopes (93.2 and 96.6% respectively). The walking paradigm demonstrates a different pattern: motion in the DOT data is coincident with MPU features in 77.8% of cases, while when no artifact is apparent in the DOT data, the rate of agreement is 47.4%. However, this is likely a result of the MPU feature extraction algorithm marking the entirety of each walking block as motion artifact.

## 4. Discussion

Advancing DOT technologies away from the use of optical fibres and towards head-mounted modular electronics has the potential to permit functional neuroimaging of the whole adult cortex, with sub-centimetre resolution, in almost any environment [30]. Dispensing with optical fibres allows for sampling density and field-of-view to be increased without the associated increase in weight and ergonomic burden. However, achieving a light-weight device that can provide high-density sampling, wide-range of source-detector separations and a large field of view with minimal cabling is a significant challenge. Here, we have presented several significant methodological and technological advances that have allowed us to create a wearable high-density DOT technology that incorporates 12 modules, and provides 1152 logical channels per wavelength. This is sufficient to cover around one third of the adult scalp. The biggest challenge in the development of this technology was achieving a reasonable frame rate while also maintaining sufficient sensitivity and dynamic range. By optimising the gain settings of the charge-to-digital converter (DDC114, Texas Instrument, USA) and focussing on the electro-magnetic shielding of the modules, we were able to achieve a sample time per measurement of 6.8 ms while also attaining exceptional sensitivity. The in-vivo-measured noise-equivalent power, which averaged 318 fW, is (as far as the authors are aware) the lowest ever reported for a CW device. The fact that the NEP was found to vary significantly with subject suggests that our measures may actually underestimate the achievable sensitivity of this device; variation between subjects implies that either the electrical noise of the environment changed day-to-day, or (more likely) the fit of the head cap affected the intensity of background light reaching the detectors during the ‘dark’ measurements. Furthermore, the NEP is inversely dependent on integration time, such that increasing the integration period to the 20 ms, for example, would yield an NEP of approximately 85 fW, but would result in a frame rate of only ∼1 Hz, which we deemed too low for functional neuroimaging because of the increased risk of aliasing of the cardiac signal.

The dual-integration strategy described in “Methods” allowed us to effectively increase the dynamic range of the device from an intrinsic value of 94.2 dB to an effective value of 142.6 dB. This ensured that we were able to successfully collect data from the shortest, 10 mm source-detector pair without saturating the DDC114. The tunable nature of the LED overlap time also allows signal quality to be optimized across different subjects. However, the fact that the minimum *τ_ovp_* value in our group was 0.1 ms (compared to a possible minimum of 0.02 ms) suggests that our large effective dynamic range was not fully exploited in this dataset. This result implies that the source power could have been increased by a factor of ∼5 without saturating the device in these subjects. However, given the high variability in optical contact losses across subjects and phantoms etc., maintaining some headroom in the device is clearly necessary.

Note that this system, like its predecessors [26,58], has undergone a range of phantom-based assessments. However, partly because building a realistic, dynamic DOT phantom that can accurately mimic the realities of an in-vivo experiment remains very challenging, we focused on assessing system performance in a real experimental scenario. One specific experimental factor that affects all optical devices is thermal stability, which is directly relevant to head-mounted electronics. The data obtained from the in-vivo experiments here did not exhibit obvious thermal drift, as the process of calibration for the integration timing scheme (which usually takes 5-10 minutes) tends to provide enough time for the modules to reach a stable temperature.

The signal quality observed in our subject group was generally high. Two subjects of 9 were rejected because the proportion of ‘good’ channels in range 0-35 mm did not exceed 70%, but in the remaining subjects the vast majority of those same channels (94.5%) passed our data quality checks. The longest separation ‘good’ channel in each subject varied from 34.8 to 56.5 mm. This compares very favourably with commercially available CW devices [7]. It is important to note, however, that in terms of the maximum obtainable source-detector distances, the performance of this system in this cohort fell short of that obtained by Chitnis et al. [26], despite the improvement in sensitivity. This is almost certainly due to the fact that the group studied here happened to include a larger proportion of subjects with dark hair.

Another significant advantage of moving DOT technologies away from the use of optical fibres and towards wearable electronics is the opportunity it presents for the integration of additional sensing devices into the HD-DOT modules. The inclusion of the motion sensor has the potential to allow automated detection and rejection, or even optimized correction, of motion-contaminated data segments [47–49,56,57]. However, our results demonstrate that using motion sensor data, even as a simple indicator of the presence of artifacts is not straightforward. While a subject remains otherwise still, our results suggest that DOT artifacts can be coincident with clear features in the MPU data, but this is not routinely the case. When a subject is undertaking a gross movement (e.g. walking), the relationship between the amplitude signal from the accelerometer and the presence of motion artifacts decouples further. The acceleration associated with walking (and in this case, turning) appear significantly larger in amplitude than the spikes in acceleration associated with motion artifacts when a subject remains still (Fig. 6), but walking itself does not necessarily induce overt motion artifacts. Extracting multi-axes data from each module, and perhaps investigating the relationship between the network of motion sensor signals that can be obtained from multiple modules, may yet provide a way of overcoming at least some of these limitations [50].

The functional haemodynamic response data obtained in this cohort was in some respects consistent with previous work and with our expectations [26,27,51,52]. The seated texting task constitutes a unimanual motor stimulation, but one that likely induces activation in the areas of the brain associated with working memory and motor planning. Given the field-of-view associated with the array, the largest responses are evident as expected in the primary somatomotor regions, specifically around the pre- and post-central gyri, approximately half way between the mid-line and the lateral cerebral fissure (Fig. 3 and 4). This area (the well-known ‘hand knob’) is invariably associated with cortical representations of the fingers and thumb [53]. The functional imaging map associated with the walking paradigm shows a response that is markedly superior to those resulting from the texting stimulus. This is consistent with a greater involvement of the cortical representation of the feet [54], and possibly of parts of the supplementary motor area [55].

As can be seen from Fig. 3, 4 and 5, the spatial distribution of the functional maps associated with the walking texting and seated texting tasks are extremely similar. The peaks of each response distribution are highly consistent for both left and right-hand stimuli. However, as evident from Fig. 4, the T-statistic maps associated with these images are very different, with a much-reduced region of activation reaching significance in the case of walking and right-hand texting, while the walking and left-hand texting fails to reach significance at all after correction. There are clearly two possible reasons for this: either the functional response that results from the texting task is reduced while the subject is also walking or the walking-texting data was simply noisier. Our analysis of the differences in motion burden and data quality across our three paradigms demonstrates that the proportion of data identified as motion in each of the three paradigms is effectively the same, but that the walking paradigms yield data that exhibits significantly larger standard deviation. This suggests there are additional features in the walking and walking texting data that are not picked up by our motion artifacts detection process, but that still increase the variance of our data. While it is possible that this increase in standard deviation during the walking tasks relates directly to physiology (e.g. increases in heart rate, stroke volume, respiration rate etc.), it is also possible that these additional features are effectively small-scale motion artifacts that do not exhibit the large, fast changes in amplitude or standard deviation that we typically depend on to identify artifacts from DOT data. The presence of these ‘micro-artifacts’ would explain the increase in noise in the walking texting haemodynamic images relative to the seated texting equivalents in Fig. 3, and the reduction in the regions of the brain in which the functional response reaches significance in Fig. 4.

The consistency of the spatial distribution of the responses between seated and walking texting is encouraging evidence that wearable HD-DOT will be able to provide reliable functional neuroimaging of subjects during overt movement, but our results suggest that improvements in head-gear and the expansion of the use of motion sensing may be necessary to obtain results equivalent to those in stationary subjects.

## 5. Conclusion

We have presented a series of significant technological developments that have allowed us to produce a fibreless, modular, high-density DOT system with exceptional sensitivity, a large dynamic range and a field of view that encompasses approximately one third of the adult scalp. We have applied this technology in a set of novel paradigms that allowed us to investigate its efficacy in the functional neuroimaging of overtly moving subjects. The system yielded comparable images of functional activation in response to a paradigm performed by both stationary and walking subjects, demonstrating the potential for this technology to become the gold-standard in un-constrained functional neuroimaging.

## Appendix 1

Figure 7 shows the full time-course of 100s of static epoxy-resin phantom data recorded using the 12-module system from cold without explicit warm-up time excepting the ∼5 minutes required to calibrate the integration timing scheme. The black dashed line denotes the noise-equivalent power, determined from the standard-deviation of dark measures. Figure 7(b) shows the temporal stability of a subset of the channels shown in Fig. 7(a) for which there was a good level of light recorded (taken here as > 1 nW). No variation exceeds 1.5%, and the median standard deviation across all channels in this example was 0.048%. Figure 7(c) shows a histogram of the gradients of the linear fit to each of the time-courses shown in b). The mean and maximum gradients were 5.8e-4% per second and 4.4e-3% per second, respectively. As the mean is non-zero, this implies there remains a linear trend present in the data, but that trend is small. For the worst-case channel, the signal intensity would expect to change by 1% in 247 seconds, while for the average channel, it would take 1700 seconds. This level of drift comparable with previous fNIRS devices [59], and is of a scale and duration that would render it irrelevant to most functional DOT paradigms.

## References

[1] van den Heuvel M. P. Hulshoff Pol H. E., “Exploring the brain network: A review on resting-state fMRI functional connectivity,” Eur. Neuropsychopharmacol. 20(8), 519–534 (2010). 10.1016/j.euroneuro.2010.03.008 20471808

[2] Weiss-Croft L. J. Baldeweg T., “Maturation of language networks in children: A systematic review of 22years of functional MRI,” NeuroImage 123, 269–281 (2015). 10.1016/j.neuroimage.2015.07.046 26213350

[3] Sperling R., “The potential of functional MRI as a biomarker in early Alzheimer's disease,” Neurobiol. Aging 32, S37–S43 (2011). 10.1016/j.neurobiolaging.2011.09.009 22078171 PMC3233699

[4] Lloyd-Fox S. Blasi A. Pasco G. Gliga T. Jones E. Murphy D. Elwell C. E. Charman T. Johnson M. H. Team B. A. S. I. S., “Cortical responses before 6 months of life associate with later autism,” Eur. J. Neurosci. 47(6), 736–749 (2018). 10.1111/ejn.13757 29057543 PMC5900943

[5] Boas D. A. Elwell C. E. Ferrari M. Taga G., “Twenty years of functional near-infrared spectroscopy: introduction for the special issue,” NeuroImage 85, 1–5 (2014). 10.1016/j.neuroimage.2013.11.033 24321364

[6] Lloyd-Fox S. Blasi A. Elwell C. E., “Illuminating the developing brain: The past, present and future of functional near infrared spectroscopy,” Neurosci. Biobehav. Rev. 34(3), 269–284 (2010). 10.1016/j.neubiorev.2009.07.008 19632270

[7] Scholkmann F. Kleiser S. Metz A. J. Zimmermann R. Pavia J. M. Wolf U. Wolf M., “A review on continuous wave functional near-infrared spectroscopy and imaging instrumentation and methodology,” NeuroImage 85, 6–27 (2014). 10.1016/j.neuroimage.2013.05.004 23684868

[8] Lloyd-Fox S. Papademetriou M. Darboe M. K. Everdell N. L. Wegmuller R. Prentice A. M. Moore S. E. Elwell C. E., “Functional near infrared spectroscopy (fNIRS) to assess cognitive function in infants in rural Africa,” Sci. Rep. 4(1), 4740 (2015). 10.1038/srep04740 PMC5381189 24751935

[9] Hoshi Y., “Functional near-infrared spectroscopy: current status and future prospects,” J. Biomed. Opt. 12(6), 062106 (2007). 10.1117/1.2804911 18163809

[10] Hoshi Y., “Functional near-infrared optical imaging: Utility and limitations in human brain mapping,” Psychophysiology 40(4), 511–520 (2003). 10.1111/1469-8986.00053 14570159

[11] Taga G. Asakawa K. Maki A. Konishi Y. Koizumi H., “Brain imaging in awake infants by near-infrared optical topography,” Proc. Natl. Acad. Sci. 100(19), 10722–10727 (2003). 10.1073/pnas.1932552100 12960368 PMC196871

[12] Cooper R. J. Selb J. Gagnon L. Phillip D. Schytz H. W. Iversen H. K. Ashina M. Boas D. A., “A Systematic Comparison of Motion Artifact Correction Techniques for Functional Near-Infrared Spectroscopy,” Front. Neurosci. 6, 147 (2012). 10.3389/fnins.2012.00147 23087603 PMC3468891

[13] Brigadoi S. Ceccherini L. Cutini S. Scarpa F. Scatturin P. Selb J. Gagnon L. Boas D. A. Cooper R. J., “Motion artifacts in functional near-infrared spectroscopy: A comparison of motion correction techniques applied to real cognitive data,” NeuroImage 85, 181–191 (2014). 10.1016/j.neuroimage.2013.04.082 23639260 PMC3762942

[14] Pinti P. Tachtsidis I. Hamilton A. Hirsch J. Aichelburg C. Gilbert S. Burgess P. W., “The present and future use of functional near-infrared spectroscopy (fNIRS) for cognitive neuroscience,” Ann. N. Y. Acad. Sci. 1464(1), 5–29 (2020). 10.1111/nyas.13948 30085354 PMC6367070

[15] Gibson A. P. Hebden J. C. Arridge S. R., “Recent advances in diffuse optical imaging,” Phys. Med. Biol. 50(4), R1–R43 (2005). 10.1088/0031-9155/50/4/R01 15773619

[16] Cooper R. J. Caffini M. Dubb J. Fang Q. Custo A. Tsuzuki D. Fischl B. Wells W. Dan I. Boas D. A., “Validating atlas-guided DOT: A comparison of diffuse optical tomography informed by atlas and subject-specific anatomies,” NeuroImage 62(3), 1999–2006 (2012). 10.1016/j.neuroimage.2012.05.031 22634215 PMC3408558

[17] Zeff B. W. White B. R. Dehghani H. Schlaggar B. L. Culver J. P., “Retinotopic mapping of adult human visual cortex with high-density diffuse optical tomography,” Proc. Natl. Acad. Sci. 104(29), 12169–12174 (2007). 10.1073/pnas.0611266104 17616584 PMC1924577

[18] Eggebrecht A. T. Ferradal S. L. Robichaux-Viehoever A. Hassanpour M. S. Dehghani H. Snyder A. Z. Hershey T. Culver J. P., “Mapping distributed brain function and networks with diffuse optical tomography,” Nat. Photonics 8(6), 448–454 (2014). 10.1038/nphoton.2014.107 25083161 PMC4114252

[19] Arridge S. Cooper R. J., “Optical Image Reconstruction in Brain Mapping,” Toga A. W., Ed., pp. 217–222, Academic Press, Waltham (2015).

[20] White B. R. Culver J. P., “Quantitative evaluation of high-density diffuse optical tomography: in vivo resolution and mapping performance,” J. Biomed. Opt. 15(2), 026006 (2010). 10.1117/1.3368999 20459251 PMC2874047

[21] Eggebrecht A. T. White B. R. Ferradal S. L. Chen C. Zhan Y. Snyder A. Z. Dehghani H. Culver J. P., “A quantitative spatial comparison of high-density diffuse optical tomography and fMRI cortical mapping,” NeuroImage 61(4), 1120–1128 (2012). 10.1016/j.neuroimage.2012.01.124 22330315 PMC3581336

[22] Dehghani H. White B. R. Zeff B. W. Tizzard A. Culver J. P., “Depth sensitivity and image reconstruction analysis of dense imaging arrays for mapping brain function with diffuse optical tomography,” Appl. Opt. 48(10), D137–D143 (2009). 10.1364/AO.48.00D137 19340101 PMC8050954

[23] Gagnon L. Perdue K. Greve D. N. Goldenholz D. Kaskhedikar G. Boas D. A., “Improved recovery of the hemodynamic response in diffuse optical imaging using short optode separations and state-space modeling,” NeuroImage 56(3), 1362–1371 (2011). 10.1016/j.neuroimage.2011.03.001 21385616 PMC3085546

[24] Brigadoi S. Cooper R. J., “How short is short? Optimum source-detector distance for short-separation channels in functional near-infrared spectroscopy,” Neurophotonics 2(2), 025005 (2015). 10.1117/1.NPh.2.2.025005 26158009 PMC4478880

[25] Gregg N. M. White B. R. Zeff B. W. Berger A. J. Culver J. P., “Brain specificity of diffuse optical imaging: improvements from superficial signal regression and tomography,” Front. Neuroenerg. 2, 14 (2010). 10.3389/fnene.2010.00014 PMC2914577 20725524

[26] Chitnis D. Cooper R. J. Dempsey L. Powell S. Quaggia S. Highton D. Elwell C. Hebden J. C. Everdell N. L., “Functional imaging of the human brain using a modular, fibre-less, high-density diffuse optical tomography system,” Biomed. Opt. Express 7(10), 4275–4288 (2016). 10.1364/BOE.7.004275 27867731 PMC5102535

[27] Laura A. D. Cooper R. J. Roque T. Correia T. Magee E. Powell S. Gibson A. P. Hebden J., “Data-driven approach to optimum wavelength selection for diffuse optical imaging,” J. Biomed. Opt. 20(1), 016003 (2015). 10.1117/1.JBO.20.1.016003 25562501

[28] Strangman G. Franceschini M. A. Boas D. A., “Factors affecting the accuracy of near-infrared spectroscopy concentration calculations for focal changes in oxygenation parameters,” NeuroImage 18(4), 865–879 (2003). 10.1016/S1053-8119(03)00021-1 12725763

[29] Gagnon L. Cooper R. J. Yücel M. A. Perdue K. L. Greve D. N. Boas D. A., “Short separation channel location impacts the performance of short channel regression in NIRS,” NeuroImage 59(3), 2518–2528 (2012). 10.1016/j.neuroimage.2011.08.095 21945793 PMC3254723

[30] Zhao H. Cooper R. J., “Review of recent progress toward a fiberless, whole-scalp diffuse optical tomography system,” Neurophotonics 5(1), 1 (2017). 10.1117/1.NPh.5.1.011012 PMC5613216 28983490

[31] Avants B. B. Tustison N. J. Song G. Cook P. A. Klein A. Gee J. C., “A reproducible evaluation of ANTs similarity metric performance in brain image registration,” NeuroImage 54(3), 2033–2044 (2011). 10.1016/j.neuroimage.2010.09.025 20851191 PMC3065962

[32] Ashburner J. Friston K. J., “Unified segmentation,” NeuroImage 26(3), 839–851 (2005). 10.1016/j.neuroimage.2005.02.018 15955494

[33] Qianqian F. Boas D. A., “Tetrahedral mesh generation from volumetric binary and grayscale images,” 2009 IEEE Int. Symp. Biomed. Imaging Nano Macro, 1142–1145 (2009).

[34] Besl P. J. McKay N. D., “A method for registration of 3-D shapes,” IEEE Trans. Pattern Anal. Mach. Intell. 14(2), 239–256 (1992). 10.1109/34.121791

[35] Aasted C. M. Yücel M. A. Cooper R. J. Dubb J. Tsuzuki D. Becerra L. Petkov M. P. Borsook D. Dan I. Boas D. A., “Anatomical guidance for functional near-infrared spectroscopy: AtlasViewer tutorial,” Neurophotonics 2(2), 020801 (2015). 10.1117/1.NPh.2.2.020801 26157991 PMC4478785

[36] Schweiger M. Arridge S. R., “The Toast++ software suite for forward and inverse modeling in optical tomography,” J. Biomed. Opt. 19(4), 040801 (2014). 10.1117/1.JBO.19.4.040801 24781586

[37] Bevilacqua F. Piguet D. Marquet P. Gross J. D. Tromberg B. J. Depeursinge C., “In vivo local determination of tissue optical properties: applications to human brain,” Appl. Opt. 38(22), 4939–4950 (1999). 10.1364/AO.38.004939 18323984

[38] Custo A. Wells W. M. Barnett A. H. Hillman E. M. C. Boas D. A., “Effective scattering coefficient of the cerebral spinal fluid in adult head models for diffuse optical imaging,” Appl. Opt. 45(19), 4747–4755 (2006). 10.1364/AO.45.004747 16799690

[39] Chiarelli A. M. Maclin E. L. Fabiani M. Gratton G., “A kurtosis-based wavelet algorithm for motion artifact correction of fNIRS data,” NeuroImage 112, 128–137 (2015). 10.1016/j.neuroimage.2015.02.057 25747916 PMC4408240

[40] Zhao H. Tanikawa Y. Gao F. Onodera Y. Sassaroli A. Tanaka K. Yamada Y., “Maps of optical differential pathlength factor of human adult forehead, somatosensory motor and occipital regions at multi-wavelengths in NIR,” Phys. Med. Biol. 47(12), 3062075 (2002). 10.1088/0031-9155/47/12/306 12118602

[41] Corlu A. Choe R. Durduran T. Lee K. Schweiger M. Arridge S. R. Hillman E. M. Yodh A. G., “Diffuse optical tomography with spectral constraints and wavelength optimization,” Appl. Opt. 44(11), 2082–2093 (2005). 10.1364/AO.44.002082 15835357

[42] Brigadoi S. Phan P. Highton D. Powell S. Cooper R. J. Hebden J. Smith M. Tachtsidis I. Elwell C. E. Gibson A. P., “Image reconstruction of oxidized cerebral cytochrome C oxidase changes from broadband near-infrared spectroscopy data,” Neurophotonics 4(2), 021105 (2017). 10.1117/1.NPh.4.2.021105 28560239 PMC5443419

[43] Glasser M. F. Sotiropoulos S. N. Wilson J. A. Coalson T. S. Fischl B. Andersson J. L. Xu J. Jbabdi S. Webster M. Polimeni J. R. Van Essen D. C. Jenkinson M. WU-Minn HCP Consortium, “The minimal preprocessing pipelines for the Human Connectome Project,” NeuroImage 80, 105–124 (2013). 10.1016/j.neuroimage.2013.04.127 23668970 PMC3720813

[44] Van Essen D. C. Glasser M. F. Dierker D. L. Harwell J. Coalson T., “Parcellations and Hemispheric Asymmetries of Human Cerebral Cortex Analyzed on Surface-Based Atlases,” Cereb. Cortex 22(10), 2241–2262 (2012). 10.1093/cercor/bhr291 22047963 PMC3432236

[45] Winkler A. M. Ridgway G. R. Webster M. A. Smith S. M. Nichols T. E., “Permutation inference for the general linear model,” NeuroImage 92, 381–397 (2014). 10.1016/j.neuroimage.2014.01.060 24530839 PMC4010955

[46] Winkler A. M. Ridgway G. R. Douaud G. Nichols T. E. Smith S. M., “Faster permutation inference in brain imaging,” NeuroImage 141, 502–516 (2016). 10.1016/j.neuroimage.2016.05.068 27288322 PMC5035139

[47] Virtanen J. Kotilahti K. M. Ilmoniemi R. Noponen T. E. J. Virtanen J., “Accelerometer-based method for correcting signal baseline changes caused by motion artifacts in medical near-infrared spectroscopy,” J. Biomed. Opt. 16(8), 087005 (2011). 10.1117/1.3606576 21895332

[48] Robertson F. C. Douglas T. S. Meintjes E. M., “Motion Artifact Removal for Functional Near Infrared Spectroscopy: A Comparison of Methods,” IEEE Trans. Biomed. Eng. 57(6), 1377–1387 (2010). 10.1109/TBME.2009.2038667 20172809

[49] Cui X. Baker J. M. Liu N. Reiss A. L., “Sensitivity of fNIRS measurement to head motion: An applied use of smartphones in the lab,” J. Neurosci. Methods 245, 37–43 (2015). 10.1016/j.jneumeth.2015.02.006 25687634 PMC4398057

[50] Brigadoi S. Ganglani A. Zhao H. Cooper R. J., “Integrating motion sensing and wearable, modular high-density diffuse optical tomography: preliminary results,” Proc. SPIE 11074, 1107405 (2019). 10.1117/12.2527197

[51] Meier J. D. Aflalo T. N. Kastner S. Graziano M. S. A., “Complex Organization of Human Primary Motor Cortex: A High-Resolution fMRI Study,” J. Neurophysiol. 100(4), 1800–1812 (2008). 10.1152/jn.90531.2008 18684903 PMC2576195

[52] Koenraadt K. L. M. Roelofsen E. G. J. Duysens J. Keijsers N. L. W., “Cortical control of normal gait and precision stepping: An fNIRS study,” NeuroImage 85, 415–422 (2014). 10.1016/j.neuroimage.2013.04.070 23631980

[53] Yousry T. A. Schmid U. D. Alkadhi H. Schmidt D. Peraud A. Buettner A. Winkler P., “Localization of the motor hand area to a knob on the precentral gyrus. A new landmark,” Brain 120(1), 141–157 (1997). 10.1093/brain/120.1.141 9055804

[54] Weiss C. Nettekoven C. Rehme A. K. Neuschmelting V. Eisenbeis A. Goldbrunner R. Grefkes C., “Mapping the hand, foot and face representations in the primary motor cortex — Retest reliability of neuronavigated TMS versus functional MRI,” NeuroImage 66, 531–542 (2013). 10.1016/j.neuroimage.2012.10.046 23116812

[55] Chainay H. Krainik A. Tanguy M. L. Gerardin E. Bihan D. L. Lehéricy S., “Foot, face and hand representation in the human supplementary motor area,” NeuroReport 15(5), 765–769 (2004). 10.1097/00001756-200404090-00005 15073511

[56] von Lühmann A. Boukouvalas Z. Müller K. R. Adalı T., “A new blind source separation framework for signal analysis and artifact rejection in functional Near-Infrared Spectroscopy,” NeuroImage 200, 72–88 (2019). 10.1016/j.neuroimage.2019.06.021 31203024

[57] von Lühmann A. Li X. Müller K. R. Boas D. A. Yücel M. A., “Improved physiological noise regression in fNIRS: A multimodal extension of the General Linear Model using temporally embedded Canonical Correlation Analysis,” NeuroImage 208, 116472 (2020). 10.1016/j.neuroimage.2019.116472 31870944 PMC7703677

[58] Chitnis D. Airantzis D. Highton D. Williams R. Phan P. Giagka V. Powell S. Cooper R. Tachtsidis I. Smith M. Elwell C. E. Hebden J. C. Everdell N., “Towards a wearable near infrared spectroscopic probe for monitoring concentrations of multiple chromophores in biological tissue in vivo,” Rev. Sci. Instrum. 87(6), 065112 (2016). 10.1063/1.4954722 27370501 PMC4957669

[59] Wyzer D. Lambercy O. Scholkmann F. Wolf M., “Wearable and modular functional near-infrared spectroscopy instrument with multidistance measurements at four wavelengths,” Neurophotonics 4(4), 041413 (2016). 10.1117/1.NPh.4.4.041413 PMC5562388 28840164

